# Postoperative Loss of Skeletal Muscle Mass Predicts Poor Survival After Gastric Cancer Surgery

**DOI:** 10.3389/fnut.2022.794576

**Published:** 2022-02-01

**Authors:** Shanjun Tan, Qiulin Zhuang, Zhige Zhang, Shuhao Li, Jiahao Xu, Junjie Wang, Yanni Zhang, Qiulei Xi, Qingyang Meng, Yi Jiang, Guohao Wu

**Affiliations:** Department of General Surgery/Shanghai Clinical Nutrition Research Center, Zhongshan Hospital, Fudan University, Shanghai, China

**Keywords:** muscle loss, gastric cancer, surgery, survival, prognosis

## Abstract

**Background:**

Skeletal muscle mass deterioration is common in gastric cancer (GC) patients and is linked to poor prognosis. However, information regarding the effect of skeletal muscle mass changes in the postoperative period is scarce. This study was to investigate the link between postoperative loss of skeletal muscle mass and survival following GC surgery.

**Methods:**

Patients who underwent GC surgery between January 2015 and December 2016 were recruited into the study. Computed tomography at L3 vertebral level was used to examine skeletal muscle index prior to surgery and about 6 months after surgery. Skeletal muscle index changes were categorized as presence or absence of ≥5% loss. Overall survival (OS) and disease-free survival (DFS) were analyzed, and Cox proportional hazard models used to identify their predictors.

**Results:**

The study comprised of 318 gastric cancer patients of which 63.5% were male. The group's mean age was 58.14 ± 10.77 years. Sixty-five patients experienced postoperative skeletal muscle index loss ≥5% and had poorer OS (*P* = 0.004) and DFS (*P* = 0.020). We find that postoperative skeletal muscle index loss ≥ 5% predicts OS [hazard ratio (HR): 2.769, 95% confidence interval (CI): 1.865–4.111; *P* < 0.001] and DFS (HR: 2.533, 95% CI: 1.753–3.659; *P* < 0.001).

**Conclusions:**

Loss of skeletal muscle mass postoperatively is linked to poor survival following GC surgery. Further studies are needed to determine whether stabilizing or enhancing skeletal muscle mass after surgery improves survival.

## Introduction

While its incidence rate continues to decrease in most parts of the world, gastric cancer (GC) accounts for the fifth common cancer and become the third leading cause of cancer-related death worldwide (1, 2). GC is mostly diagnosed after it has progressed to an advanced stage, and as such, has a low 5-year survival (3). Surgical resection is the most effective therapeutic intervention against GC (4). However, despite advances in operative techniques and perioperative care, GC prognosis after surgery remains poor (5). Numerous studies have shown that cancer prognosis is conditioned not only by non-modifiable tumor-specific factors such as histology and stage but also modifiable patient-individual factors such as performance status (i.e., patients' physical functioning associated with activities of daily life) and body composition (6–8). Thus, timely identification of these modifiable factors is needed for effective targeted interventions and improved prognosis.

Examination of body composition and its influence on cancer outcomes has drawn growing interest in surgical oncology. Notably, loss of skeletal muscle mass has cancer prognostic value (6–8). Preoperative reduction in skeletal muscle mass is related to poor prognosis after surgical treatment of various cancers, including GC (9–11). Identifying skeletal muscle mass preoperative loss is prognostic and may allow timely therapeutic intervention for better GC outcomes. However, GC patients are often malnourished before surgery, and their malnutrition is often worsened by various factors like postoperative chemotherapy and reduced stomach volume (12). Significant postoperative weight loss has been reported after GC surgery (13, 14), suggesting that muscle wasting might occur in the postoperative period. We have recently reported that after GC surgery, reduced skeletal muscle mass occurs in 3 months after hospital discharge (15). Postoperative skeletal muscle mass has also been reported to negatively impact survival after digestive tract cancer surgeries, including pancreatic, colorectal, and esophageal cancer (7, 16, 17). However, as far as we know, earlier studies mainly concentrated on the influence of preoperative skeletal muscle mass loss on postsurgical GC prognosis. Thus, it is unclear whether the loss of skeletal muscle postoperatively is a risk factor for poor GC prognosis after surgery. If this were the case, then serial assessment of skeletal muscle mass postoperatively may guide efficient interventions.

Here, we aimed to assess postoperative changes in skeletal muscle mass using computed tomography (CT) after GC surgery and to determine whether these changes affect overall and disease-free survival.

## Materials and Methods

### Study Population

Patients aged > 18 years, who underwent GC surgery between January 2015 and December 2016 at the Department of General Surgery/Shanghai Clinical Nutrition Research Center, Zhongshan Hospital, Fudan University, China, were recruited into the study from our prospective clinical database. Patients under palliative or emergency surgery were excluded from the study. Our institutional ethics committee provided ethical approval for the study, which was conducted based on the Declaration of Helsinki ethical standards.

### Assessment of Skeletal Muscle Mass

We utilized routine patient abdominal CT scans to examine skeletal muscle mass, as we previously described (18). The CT images used were either contrast-enhanced or unenhanced multiphase acquisitions, 5 mm thick. Two adjacent CT images at L3 vertebral levels in the same series were chosen in the non-contrast phase. Next, total skeletal muscle area (SMA) was quantified using ImageJ2 software (The National Institutes of Health, Washington, MD, USA) between −29 to +150 Hounsfield units (HU) for skeletal muscle on both slices, and the average SMA reported. Skeletal muscle index (SMI) was computed using the formula: SMI = SMA/height^2^, expressed in cm^2^/m^2^. Anonymized CT images were analyzed by an experienced study evaluator who was not aware of the order of images. All included patients underwent abdominal CT scans within 7 days before surgery and about 6 months after surgery, and SMI changes were calculated. Because skeletal muscle losses ≥ 5% have previously been associated with poor clinical outcomes, including short survival in cancer treatment (19), we used this cutoff threshold to define the postoperative loss of skeletal muscle mass by grouping patients as SMI loss ≥ 5% or SMI loss < 5%.

### Data Collection

The clinical data collected included demographics, preoperative characteristics [including BMI (body mass index), ECOG (eastern cooperative oncology group) performance status, serum hemoglobin and albumin level, and comorbidities], operative and pathologic features [including tumor location, type of resection, type of reconstruction, histology, and cancer stage based on the 8th AJCC (American joint committee on cancer) edition], postoperative characteristics (postoperative hospital stay, postoperative complications examined based on the Dindo and Clavien classification), number of patients needing chemotherapy, and chemotherapy tolerance (defined as chemotherapy modification including dose reduction, delay, or termination, and evaluated using a dichotomous scale of absent vs. present) (20). Data on overall survival (OS) and disease-free survival (DFS) were collected. In our prospective clinical database, the follow-up period for all patients was 1^st^-month post-surgery and after every 3 months, until June 2020.

### Statistical Analysis

Statistical analysis was done on SPSS 23.0 software (SPSS Inc., Chicago, IL, USA). Continuous data are expressed as mean ± standard deviation (SD), whereas categorical data are shown as percentages and numbers. Independent-samples *t*-test or Mann-Whitney *U* test was employed to analyze continuous variables. χ^2^ test or the Fisher exact test was used to compare categorical data. Kaplan-Meier analyses were used to generate OS and DFS curves. Variations in survival were analyzed using the log-rank test. The impact of postoperative skeletal muscle mass loss on survival was investigated using Cox proportional hazard models. First, univariate analyses were performed respectively for all potential variables that were chosen based on clinical information. Multivariate analysis was then done using Cox proportional backward stepwise procedure, including all variables with *P* < 0.05 in the univariate analysis. *P* < 0.05 indicates statistical significance.

## Results

### Patient Characteristics

Of the 363 patients who consecutively underwent curative GC surgery from January 2015 to December 2016, 318 patients (63.5% male, mean age 58.14 years) met the inclusion criteria. 65 patients exhibited SMI losses ≥ 5%, while 253 had SMI losses < 5%. Participant characteristics are shown on Table 1. The groups with ≥ 5% SMI loss and the one with < 5% SMI loss were similar with regards to gender, diabetes, respiratory and cardiovascular comorbidity, serum albumin and hemoglobin, preoperative BMI, preoperative SMI, preoperative ECOG performance status, tumor location, type of resection, type of reconstruction, histology, and postoperative hospital stay (*P* > 0.05). However, ≥ 5% loss significantly correlated with advanced age (60.86 ± 10.73 vs. 57.45 ± 10.68 years, *P* = 0.022), higher incidence of postoperative complications (26.2 vs. 15.4%; *P* = 0.043), higher rates of postoperative chemotherapy (78.5 vs. 65.2%; *P* = 0.041), and chemotherapy modification including dose reduction, delay, or termination (35.4 vs. 19.8%; *P* = 0.008). Moreover, AJCC stage differed significantly between the two groups (*P* = 0.010).

**Table 1:** Patient demographic and clinical characteristics according to postoperative skeletal muscle mass loss.

**Characteristics**	**Total** **(***n*** = 318)**	**SMI loss ≥5%** **(***n*** = 65)**	**SMI loss < 5%** **(***n*** = 253)**	***P*** **-value**
Gender				0.284
Male Female	202 (63.5) 116 (36.5)	45 (69.2) 20 (30.8)	157 (62.1) 96 (37.9)	
**Age (years), mean** **±SD**	58.14 ± 10.77	60.86 ± 10.73	57.45 ± 10.68	**0.022**
Diabetes	21 (6.6)	6 (9.2)	15 (5.9)	0.399
Respiratory comorbidity	17 (5.3)	3 (4.6)	14 (5.5)	1.000
Cardiovascular comorbidity	58 (18.2)	13 (20)	45 (17.8)	0.680
Serum albumin (g/L), mean ± SD	38.46 ± 4.79	37.54 ± 4.47	38.69 ± 4.85	0.083
Serum hemoglobin (g/L), mean ± SD	122.72 ± 23.58	119.46 ± 21.77	123.56 ± 23.99	0.212
Preoperative BMI (kg/m^2^), mean ± SD	22.29 ± 3.38	21.61 ± 3.29	22.47 ± 3.38	0.067
Preoperative SMI (cm^2^/m^2^), mean ± SD	42.60 ± 5.23	41.71 ± 5.34	42.82 ± 5.18	0.124
Preoperative ECOG performance status				0.549
0 1	261 (82.1) 57 (17.9)	55 (84.6) 10 (15.4)	206 (81.4) 47 (18.6)	
Tumor location				0.816
Upper Not upper	70 (22.0) 248 (78.0)	15 (23.1) 50 (76.9)	55 (21.7) 198 (78.3)	
Type of resection				0.596
Total gastrectomy Subtotal gastrectomy	99 (31.1) 219 (68.9)	22 (33.8) 43 (66.2)	77 (30.4) 176 (69.6)	
Type of reconstruction				0.741
Billroth I Billroth II Roux-en-Y Other	121 (38.1) 69 (21.7) 117 (36.8) 11 (3.5)	26 (40.0) 11 (16.9) 26 (40.0) 2 (3.1)	95 (37.5) 58 (22.9) 91 (36.0) 9 (3.6)	
Histology				0.793
Undifferentiated Differentiated	113 (35.5) 205 (64.5)	24 (36.9) 41 (63.1)	89 (35.2) 164 (64.8)	
**AJCC stage**				**0.010**
I II III	79 (24.8) 115 (36.2) 124 (39.0)	12 (18.5) 17 (26.2) 36 (55.4)	67 (26.5) 98 (38.7) 88 (34.8)	
**Postoperative any complication**	56 (17.6)	17 (26.2)	39 (15.4)	**0.043**
Postoperative hospital stay (days), mean ± SD	9.48 ± 2.17	9.72 ± 2.50	9.42 ± 2.08	0.314
**Postoperative chemotherapy**	216 (67.9)	51 (78.5)	165 (65.2)	**0.041**
**Chemotherapy modification**	73 (23.0)	23 (35.4)	50 (19.8)	**0.008**

*Values are presented as n (%) unless otherwise stated. Bold values indicate statistical significant*.

*SD, standard deviation; BMI, body mass index; SMI, skeletal muscle index; AJCC, American Joint Committee on Cancer; ECOG, Eastern Cooperative Oncology Group*.

### Effects of Postoperative Skeletal Muscle Mass Loss on Overall Survival

**Figure 1:**
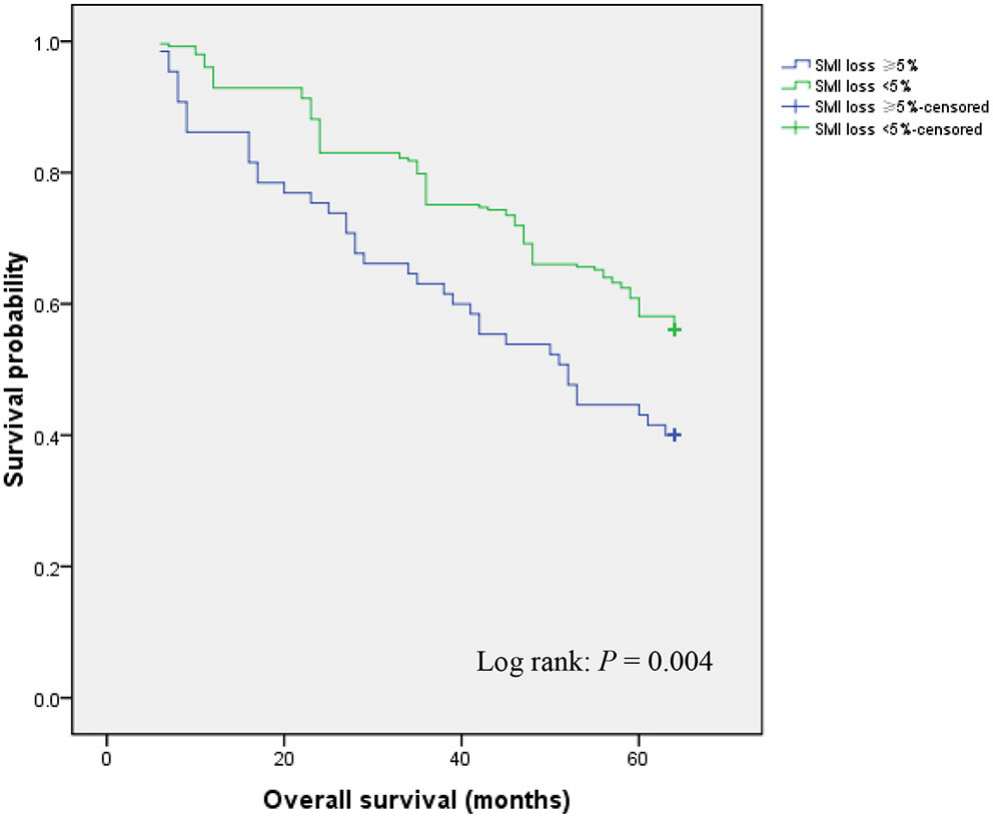
Overall survival according to postoperative skeletal muscle mass loss.

During follow-up, patients who exhibited ≥ 5% SMI loss showed significantly lower OS relative to those with < 5% SMI loss (40.0 vs. 56.1%; *P* = 0.004) (Figure 1). Univariate and multivariate analyses were used to identify factors influencing OS following GC curative surgery (Table 2). Univariate analysis revealed the following factor as significantly-associated with poor OS: age ≥ 65 years [hazard ratio (HR) = 1.638, 95% confidence interval (CI) = 1.160–2.314; *P* = 0.005], hypoproteinemia (HR = 1.501, 95% CI = 1.035–2.328; *P* = 0.043), preoperative SMI (HR = 2.546, 95% CI = 1.774–3.653; *P* < 0.001), histology (HR = 1.500, 95% CI = 1.083–2.078; *P* = 0.015), AJCC stage (II vs. I: HR = 6.355, 95% CI = 3.719–10.859; *P* < 0.001; III vs. I: HR = 6.930, 95% CI = 4.200–11.435; *P* < 0.001), postoperative any complication (HR = 1.494, 95% CI = 1.011–2.209; *P* = 0.044), postoperative chemotherapy (HR = 1.619, 95% CI = 1.117–2.348; *P* = 0.011), chemotherapy modification (HR = 1.545, 95% CI = 1.081–2.207; *P* = 0.017), and SMI loss ≥ 5% (HR = 1.693, 95% CI = 1.175–2.439; *P* = 0.005). Multivariate analysis identified the following factors as independently correlating with poor OS: age ≥65 years (HR = 1.616, 95% CI = 1.130–2.311; *P* = 0.009), preoperative SMI (HR = 2.187, 95% CI = 1.491–3.208; *P* < 0.001), AJCC stage (II vs. I: HR = 6.106, 95% CI = 3.504–10.641; *P* < 0.001; III vs. I: HR = 8.840, 95% CI = 5.231–14.938; *P* < 0.001), chemotherapy modification (HR = 1.498, 95% CI = 1.079–2.325; *P* = 0.032), and SMI loss ≥ 5% (HR = 2.769, 95% CI = 1.865–4.111; *P* < 0.001).

**Table 2:** Univariate and multivariate analyses of prognostic factors for overall survival.

	**Univariate analysis**		**Multivariate analysis**	
	**HR (95% CI)**	***P*** **-value**	**HR (95% CI)**	***P*** **-value**
Gender				
Male vs. female	1.118 (0.763–1.639)	0.567		
**Age, years**				
≥ 65 vs. < 65	1.638 (1.160–2.314)	0.005	1.616 (1.130–2.311)	**0.009**
Diabetes				
Yes vs. no	1.572 (0.890–2.777)	0.120		
Respiratory comorbidity				
Yes vs. no	1.329 (0.587–3.008)	0.495		
Cardiovascular comorbidity				
Yes vs. no	1.066 (0.714–1.591)	0.754		
Hypoproteinemia				
Yes vs. no	1.501 (1.035–2.328)	0.043	1.432 (0.975–1.072)	0.097
Anemia				
Yes vs. no	1.188 (0.783–1.802)	0.417		
Preoperative BMI, kg/m^2^				
< 18.5 vs. 18.5–25 > 25 vs. 18.5–25	1.138 (0.704–1.839) 1.052 (0.700–1.582)	0.598 0.808		
**Preoperative SMI, cm** ^**2**^ **/m** ^**2**^				
< 43.13 for men or < 37.81 for women vs. ≥ 43.13 for men or ≥ 37.81 for women^a^	2.546 (1.774–3.653)	< 0.001	2.187 (1.491–3.208)	**< 0.001**
Preoperative ECOG performance status				
1 vs. 0	1.223 (0.887–1.687)	0.220		
Tumor location				
Upper vs. not upper	1.066 (0.721–1.575)	0.750		
Type of resection				
Total vs. subtotal	1.230 (0.874–1.730)	0.235		
Histology				
Undifferentiated vs. differentiated	1.500 (1.083–2.078)	0.015	1.098 (0.773–1.559)	0.601
**AJCC stage**				
II vs. I III vs. I	6.355 (3.719–10.859) 6.930 (4.200–11.435)	<0.001 <0.001	6.106 (3.504–10.641) 8.840 (5.231–14.938)	**< 0.001** **< 0.001**
Postoperative any complication				
Yes vs. no	1.494 (1.011–2.209)	0.044	1.193 (0.797–1.786)	0.390
Postoperative chemotherapy				
Yes vs. no	1.619 (1.117–2.348)	0.011	1.229 (0.823–1.836)	0.314
**Chemotherapy modification**				
Yes vs. no	1.545 (1.081–2.207)	0.017	1.498 (1.079–2.325)	**0.032**
**SMI loss**				
≥ 5% vs. < 5%	1.693 (1.175–2.439)	0.005	2.769 (1.865–4.111)	**< 0.001**

*Bold values indicate statistical significant*.

*HR, hazard ratio; CI, confidence interval; BMI, body mass index; SMI, skeletal muscle index; ECOG, Eastern Cooperative Oncology Group*.

a *This cut point was based on the recent study showing that SMI <43.13 cm^2^/m^2^ for men or <37.81 cm^2^/m^2^ for women was associated with poor surgical and oncologic outcomes after gastrointestinal cancer surgery (18)*.

### Effects of Postoperative Loss of Skeletal Muscle Mass on Disease-Free Survival

**Figure 2:**
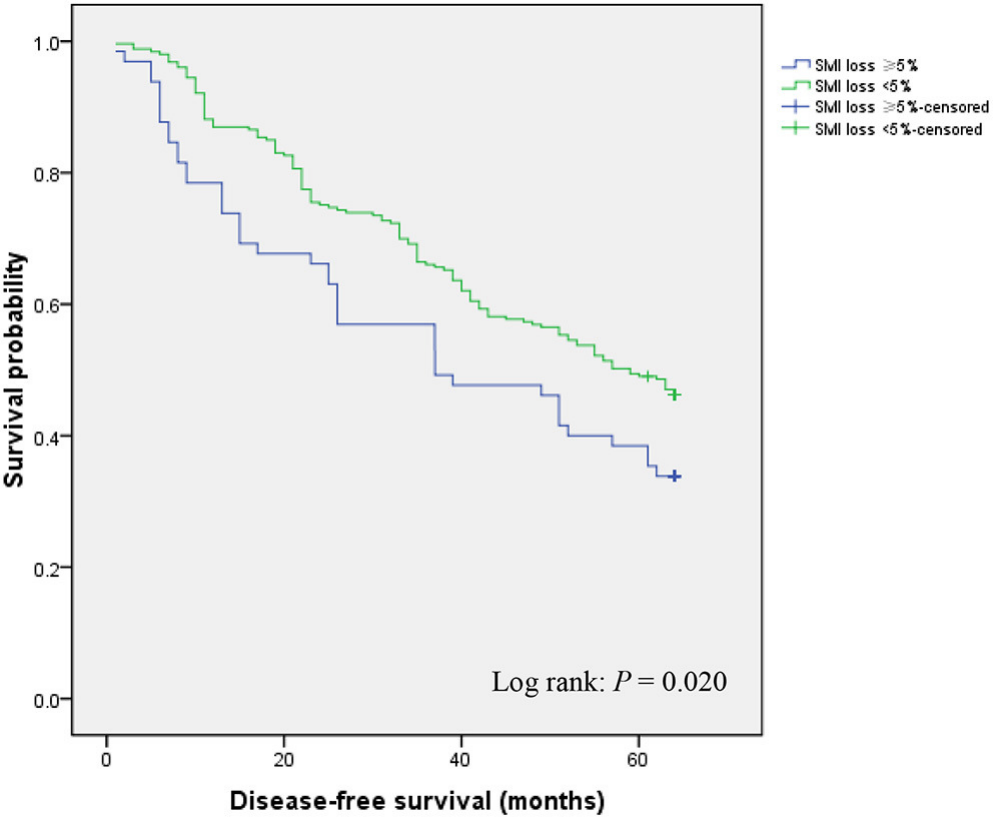
Disease-free survival according to postoperative skeletal muscle mass loss.

In the course of follow-up, patients who exhibited ≥ 5% SMI loss showed considerably lower DFS rates relative to those with < 5% SMI loss (33.8 vs. 46.2%; *P* = 0.020) (Figure 2). Univariate and multivariate analyses were used to identify factors influencing DFS following GC curative surgery (Table 3). Univariate analysis revealed the following factors as significantly correlating with poor DFS: hypoproteinemia (HR = 1.401, 95% CI = 1.022–1.922; *P* = 0.036), preoperative SMI (HR = 2.348, 95% CI = 1.675–3.290; *P* < 0.001), histology (HR = 1.774, 95% CI = 1.319–2.388; *P* < 0.001), AJCC stage (II vs. I: HR = 12.511, 95% CI = 7.524–20.804); *P* < 0.001; III vs. I: HR = 8.525, 95% CI = 5.237–13.878; *P* < 0.001), postoperative any complication (HR = 1.854, 95% CI = 1.307–2.629; *P* = 0.001), chemotherapy modification (HR = 1.513, 95% CI = 1.032–1.975; *P* = 0.019), and ≥ 5% SMI loss (HR = 1.492, 95% CI = 1.058–2.102; *P* = 0.022). Multivariate analysis identified preoperative SMI (HR = 1.953, 95% CI = 1.369–2.786; *P* < 0.001), AJCC stage (II vs. I: HR = 11.726, 95% CI = 6.983–19.690; *P* < 0.001; III vs. I: HR = 10.096, 95% CI = 6.091–16.735; *P* < 0.001), chemotherapy modification (HR = 1.403, 95% CI = 1.006–1.879; *P* = 0.041), and SMI loss ≥ 5% (HR = 2.533, 95% CI = 1.753–3.659; *P* < 0.001) as independently correlated with poor DFS.

**Table 3:** Univariate and multivariate analyses of prognostic factors for disease-free survival.

	**Univariate analysis**		**Multivariate analysis**	
	**HR (95% CI)**	***P*** **-value**	**HR (95% CI)**	***P*** **-value**
Gender				
Male vs. female	1.217 (0.861–1.721)	0.265		
Age, years				
≥ 65 vs. < 65	1.327 (0.679–1.875)	0.158		
Diabetes				
Yes vs. no	1.641 (0.967–2.785)	0.067		
Respiratory comorbidity				
Yes vs. no	1.358 (0.717–2.570)	0.348		
Cardiovascular comorbidity				
Yes vs. no	1.045 (0.722–1.512)	0.816		
Hypoproteinemia				
Yes vs. no	1.401 (1.022–1.922)	0.036	1.225 (0.879–1.709)	0.231
Anemia				
Yes vs. no	1.371 (0.926–2.0292)	0.115		
Preoperative BMI, kg/m^2^				
< 18.5 vs. 18.5–25 > 25 vs. 18.5–25	1.021 (0.648–1.609) 0.994 (0.685–1.442)	0.928 0.975		
**Preoperative SMI, cm** ^**2**^ **/m** ^**2**^				
< 43.13 for men or < 37.81 for women vs. ≥ 43.13 for men or ≥ 37.81 for women^a^	2.348 (1.675–3.290)	<0.001	1.953 (1.369–2.786)	**< 0.001**
Preoperative ECOG performance status				
1 vs. 0	1.111 (0.827–1.493)	0.485		
Tumor location				
Upper vs. not upper	1.063 (0.745–1.516)	0.735		
Type of resection				
Total vs. subtotal	1.316 (0.964–1.794)	0.083		
Histology				
Undifferentiated vs. differentiated	1.774 (1.319–2.388)	<0.001	1.307 (0.962–1.776)	0.087
**AJCC stage**				
II vs. I III vs. I	12.511 (7.524–20.804) 8.525 (5.237–13.878)	<0.001 <0.001	11.726 (6.983–19.690) 10.096 (6.091–16.735)	**< 0.001** **< 0.001**
Postoperative any complication				
Yes vs. no	1.854 (1.307–2.629)	0.001	1.371 (0.954–1.970)	0.088
Postoperative chemotherapy				
Yes vs. no	1.314 (0.948–1.822)	0.101		
**Chemotherapy modification**				
Yes vs. no	1.513 (1.032–1.975)	0.019	1.403 (1.006–1.879)	**0.041**
**SMI loss**				
≥ 5% vs. < 5%	1.492 (1.058–2.102)	0.022	2.533 (1.753–3.659)	**< 0.001**

*Bold values indicate statistical significant*.

*HR, hazard ratio; CI, confidence interval; BMI, body mass index; SMI, skeletal muscle index; ECOG, Eastern Cooperative Oncology Group*.

a *This cut point was based on the recent study showing that SMI <43.13 cm^2^/m^2^ for men or <37.81 cm^2^/m^2^ for women was associated with poor surgical and oncologic outcomes after gastrointestinal cancer surgery (18)*.

## Discussion

To our best knowledge, this study was the first report suggesting that postoperative loss of skeletal muscle mass negatively influences OS and DFS in patients following GC surgery. These findings may guide clinicians on the optimal use of prophylactic strategies to reduce postoperative skeletal muscle mass loss, aiming to improve GC outcomes after surgery.

Even though there has been a significant advancement in nutritional support therapy, surgical techniques, and increased recovery rates following surgery, GC surgery is still associated with high malnutrition risk as a result of gastrointestinal complications and reduced food intake. These problems are exacerbated by chronic comorbidities, unintentional weight loss prior to surgery, and postoperative chemotherapy (12). Poor nutrition is linked to poor clinical outcomes, which mainly include increased morbidity and mortality, as well as decreased survival (21–23). Thus, the management of malnutrition is critical for GC treatment and prognosis. Recently, loss of skeletal muscle mass emerged as a prognostic indicator in various cancers during surgery (6–11). However, studies conducted previously primarily focused on the effects of preoperative skeletal muscle mass loss after gastric cancer surgery, and it is unclear whether postoperative skeletal muscle mass loss affects post-GC surgery prognosis. Additionally, postoperative skeletal muscle mass loss negatively impacts survival after digestive tract surgery due to the pancreatic, colorectal, and esophagus cancers (7, 16, 17). Here, we sought to examine skeletal muscle mass postoperative changes after GC surgery and to determine whether these changes affect OS and DFS.

A standard technique for measuring skeletal muscle mass is lacking. Different methods like dual-energy X-ray absorptiometry and CT scanning, are applied to quantitatively measure skeletal muscle mass in clinical practice and research (24). Of the widely used techniques, CT scan has emerged as a reliable method of skeletal muscle mass measurement (25–27). Cross-sectional areas of skeletal muscle tissue on single CT slices at L3 vertebral levels have been shown to strongly correlate with total body skeletal muscle tissue. CT images provide objective quantitative measures of skeletal muscle mass via SMI calculation (28–30). Thus, the assessment of skeletal muscle mass using CT scan at L3 vertebral levels combined with SMI calculation is increasingly used to examine the impact of preoperative skeletal muscle mass changes on clinical outcomes after digestive tract cancer surgery (18, 31, 32). Here, we used CT scan to measure skeletal muscle mass before and approximately 6 months after surgery and calculated SMI changes. We considered ≥ 5% skeletal muscle loss indicative of significant postoperative skeletal muscle mass loss, since it has been previously associated with poor clinical outcomes, including short survival with cancer treatment. Our data revealed 65 of 318 patients as having ≥ 5% SMI loss in the 6 months after GC surgery. ≥ 5% SMI loss significantly correlated with older age, higher incidence of postoperative complications, and higher postoperative chemotherapy and chemotherapy modification (like dose reduction, delay/termination). There was a significant difference in AJCC stage between the two groups. However, the ≥ 5% SMI loss group was comparable to the < 5% SMI loss group with regards to gender, diabetes, respiratory and cardiovascular comorbidity, serum albumin and hemoglobin, preoperative BMI, preoperative SMI, preoperative ECOG performance status, tumor location, type of resection, type of reconstruction, histology, and postoperative hospital stay. These indicate that the ≥ 5% SMI loss criteria used in this study represents significant postoperative skeletal muscle mass loss after GC surgery. Moreover, skeletal muscle mass measurement using abdominal CT scans can be employed to postoperatively evaluate patients, as abdominal CT scans are regularly used, inexpensive, and easy to execute during follow up after GC surgery.

Regarding the survival following cancer surgery, it always receives a significant concern for the prognostic gain following oncologic surgery. Studies have mainly evaluated the association between skeletal muscle mass loss and survival postoperatively. Here, we primarily assessed the effect of postoperative skeletal muscle mass loss on OS and DFS after GC surgery. Our data show that postoperative skeletal muscle mass loss significantly correlates with lower OS and DFS following GC surgery. Multivariate analyses reveal that it is an unfavorable prognostic indicator of disease-free survival. These findings are consistent with previous reports on surgical treatment of other gastrointestinal cancers (7, 16, 17, 33, 34), indicating independent relationship between skeletal muscle mass loss postoperatively and cancer endpoints. Although we did not examine the reasons underlying the strong link between postoperative skeletal muscle mass loss and survival, we speculate that it may be due to multiple factors, including poor tolerability of systemic chemotherapy. Previous studies, including our recent one on digestive cancer surgery, show that loss of skeletal muscle mass may reduce the ability to tolerate systemic chemotherapy. Thus, patients exhibiting low skeletal muscle mass are more likely to experience extreme treatment-associated toxicities, leading to fewer completed chemotherapy cycles (18, 26, 35). Here, postoperative skeletal muscle mass loss was related to more chemotherapy modifications, like dose reduction, delay/termination, and was identified as a risk factor for poor OS and DFS following GC surgery. This could lead to poorer disease control and low survival. Nevertheless, these findings highlight the importance of identifying skeletal muscle mass loss after surgery because it allows prophylactic strategies including the use of proper nutritional support therapy and physical exercise aiming to reduce postoperative skeletal muscle mass loss.

We acknowledge the following limitations in our study. First, our analysis did not examine nutritional intake and physical activity, which are linked to skeletal muscle mass and may affect survival (36). The inclusion of these data would more comprehensively highlight the causal link between skeletal muscle mass loss and poor survival. Secondly, being a single-center study, it may exaggerate the impact of postoperative skeletal muscle mass loss on survival. Thus, there is a need to conduct international multicenter studies to verify these findings. Thirdly, recent evidence indicates that both low skeletal muscle mass and decreased skeletal muscle function influence clinical outcomes (37, 38). However, our study did not capture data on skeletal muscle functions like grip strength/walking speed because of the retrospective design of the study cohort. Further research should evaluate both skeletal muscle mass and function, to evidently reveal the effect of skeletal muscle changes on cancer patients post-surgery. Finally, there were apparent differences in participant characteristics between the two groups, such as AJCC stage, which may affect OS and DFS. The Propensity Score Matching will be conducted to comprehensively answer the question regarding the impact of postoperative loss of skeletal muscle mass on survival after GC surgery. In addition, the univariate and multivariate analyses of risk factors affecting OS and DFS in our study are of great significance, and due to the confounding factors, the significance of OS and DFS in patients with different skeletal muscles is unclear and unreliable. Thus, we will include the related analysis in our future studies in this field of research.

In this study, our data show that postoperative skeletal muscle mass loss negatively affects survival and that it has a strong, independent, prognostic value after GC surgery. Identification of postoperative skeletal muscle mass loss by abdominal CT imaging after GC surgery and targeted approaches to reduce postoperative skeletal muscle mass loss may improve GC outcomes.

## Data Availability Statement

The raw data supporting the conclusions of this article will be made available by the authors, without undue reservation.

## Ethics Statement

The studies involving human participants were reviewed and approved by Zhongshan Hospital Fudan University. The patients/participants provided their written informed consent to participate in this study.

## Author Contributions

GW supervised the entire project and ST designed the study. QZ, ZZ, SL, JX, JW, YZ, QX, QM, and YJ performed data collection. QZ and ZZ conducted data analyses. ST wrote and revised the manuscript. All authors critically reviewed and approved the final manuscript.

## Funding

This study was sponsored by Clinical Research Special Fund of Zhongshan Hospital, Fudan University (2020ZSLC17) and Construction Program of Key but Weak Disciplines of Shanghai Health Commission-Clinical Nutrition (2019ZB0105).

## Conflict of Interest

The authors declare that the research was conducted in the absence of any commercial or financial relationships that could be construed as a potential conflict of interest.

## Publisher's Note

All claims expressed in this article are solely those of the authors and do not necessarily represent those of their affiliated organizations, or those of the publisher, the editors and the reviewers. Any product that may be evaluated in this article, or claim that may be made by its manufacturer, is not guaranteed or endorsed by the publisher.
